# Factors associated with COVID-19 infection in pregnant women: Focusing on maternal anxiety

**DOI:** 10.1371/journal.pone.0312300

**Published:** 2024-10-24

**Authors:** Wonjeong Jeong, Bora Kim, Shin Hee Hong, Eunsang Cho, Suk Young Kim, Jong Youn Moon

**Affiliations:** 1 National Cancer Control Institute, Cancer Knowledge & Information Center, National Cancer Center, Goyang, Republic of Korea; 2 Center for Public Health, Gachon University Gil Medical Center, Incheon, Republic of Korea; 3 Department of Literature and Art Therapy, Konkuk University, Seoul, Republic of Korea; 4 Department of Infectious Medicine, Gachon University of Gil Medical Center, Incheon, Republic of Korea; 5 Department of Obstetrics and Gynecology, Gachon University of Gil Medical Center, Incheon, Republic of Korea; 6 Department of Preventive Medicine, Gachon University College of Medicine, Incheon, Republic of Korea; United Arab Emirates University, UNITED ARAB EMIRATES

## Abstract

**Objective:**

Given the critical importance of maternal mental health for the well-being of both the mother and fetus, it is essential to thoroughly investigate the impact of COVID-19 infection on mental health. This study aims to identify the factors associated with COVID-19 infection and mental health, underscoring the necessity of effective mental health management for pregnant women.

**Methods:**

Data were analyzed from 97 pregnant women who visited Gachon University Gil hospital in South Korea. Coronavirus 2019 (COVID-19) infection status was categorized based on whether the infection occurred during pregnancy. Maternal anxiety, the dependent variable, was measured using the state anxiety scale of the Spielberger State-Trait Anxiety Inventory. Multiple logistic regression analysis was performed to examine the association between COVID-19 infection and anxiety.

**Results:**

Among the 97 pregnant women, 50 (51.5%) experienced anxiety. Of those infected with COVID-19, 31 (64.6%) experienced anxiety. The mean anxiety score was significantly higher in pregnant women with COVID-19 infection compared to those without infection (Infected: Mean = 46.35, SD = 13.85; Non-infected: Mean = 39.59, SD = 10.58, p-value:0.008). Maternal depression, and posttraumatic stress disorder were significantly higher in pregnant women with COVID-19 infection, while fetal attachment showed no significant difference. Pregnant women infected with COVID-19 during pregnancy were more likely to experience anxiety compared to non-infected women (Adjusted OR = 9.37; 95% CI = 1.39–63.12).

**Conclusion:**

This study highlights that pregnant women infected with COVID-19 are more likely to experience elevated levels of anxiety, emphasizing the critical importance of addressing mental health among pregnant women. The insights from this study could provide valuable guidance for policymaking, underscoring the need for targeted interventions to manage mental health in pregnant women and mitigate the risk of adverse outcomes.

## Introduction

The novel coronavirus 2019 (COVID-19) has precipitated a global health crisis with significant economic and social repercussions. During pandemic outbreaks, healthcare utilization often decreases, which can adversely affect population health [1]. The COVID-19 pandemic has induced substantial stress, characterized by widespread confusion, income and housing loss, social/physical distancing, and fear of infection, all of which can contribute to changes in health symptoms [2]. As the world gradually gains control over COVID-19, it is both timely and essential to examine how the pandemic has impacted global mental health [3].

The COVID-19 pandemic has significantly disrupted the provision of maternal and neonatal health services. Social distancing measures have restricted access to these services, and many individuals have been hesitant to visit hospitals due to fears of infection [4]. Concerns about contracting COVID-19 have led people to limit daily activities such as social interactions, exercise, and medical visits [5]. As a results, managing both physical and mental health of pregnant women has become increasingly challenging.

Depression and anxiety are prevalent mood disorders affecting approximately one in seven pregnant women, and during the COVID-19 pandemic, this population has been identified as particularly vulnerable [6]. The pregnancy and postpartum periods, especially for the first-time mothers, are marked by significant social, psychological, and physiological changes [7]. The uncertainty surrounding the impact of COVID-19 on maternal and neonatal health has further heightened anxiety among pregnant women. Compromised mental health can have both immediate and long-term consequences for both mothers and their infants, underscoring the urgent need for targeted care and intervention [8].

Therefore, it is essential to investigate the factors associated with COVID-19 infection and maternal anxiety to highlight the importance of managing mental health in pregnant women. Given the critical role of maternal health for both mothers and their fetuses, a comprehensive investigation is needed to understand how COVID-19 infection impacts various mental health dimensions, including depression, maternal-fetal attachment, and posttraumatic stress disorder (PTSD), with a particular focus on maternal anxiety. This study hypothesizes that COVID-19 infection adversely affects maternal mental health, specifically increasing anxiety. Consequently, this study aims to evaluate the effects of COVID-19 infection on anxiety among pregnant women in South Korea.

## Methods

### Data and study participants

The study was conducted with pregnant women visiting the Obstetrics and Gynecology department at Gachon University Gil Hospital, located in Incheon, a metropolitan area in South Korea. The metropolitan area, comprising Seoul, Gyeonggi, and Incheon, reported the highest number of COVID-19 cases, according to sex, age, and region [9]. Gachon University Gil Hospital is a leading tertiary general hospital in Incheon. The study included pregnant women attending the hospital for routine obstetrics and gynecology checkups who were cognitively able to communicate and respond to surveys. All participants voluntarily took part in the study, providing informed consent after receiving comprehensive information about the study. Participants were blinded (single-blind), while data collectors were not. Surveys for pregnant women with COVID-19 were conducted after their medical and physical symptoms had subsided to minimize the impact of COVID-19-related anxiety and physical symptoms on the evaluation of psychological symptoms. The survey items were translated into Korean and administered by nurses familiar with the survey content. Additionally, three trained nurses, well-acquainted with the survey items, were involved in data collection. It should be noted, however, that non-blinded data collectors reported a significantly more beneficial effect of the clinical intervention compared to blinded assessors [10].

Subjects were recruited from November 9, 2021, following IRB approval, until October 7, 2022, over a period of approximately one year. The sample size for this study was determined using G*power 3.1, with the following parameters: an independent sample t-test, effect size (*f*) estimated from a prior study, two participants groups, a significance level of *α* = 0.05, and a power of 1-*β* = 0.80 [11]. Based on these assumptions, a minimum sample size of 82 participants was required, with approximately 41 participants per group. To account for a 20% dropout rate, an initial target of 100 participants was set, consisting of 50 COVID-19 infected pregnant women and 50 non-infected pregnant women. Ultimately, 3 individuals declined participation, resulting in a final sample of 48 infected pregnant women and 49 non-infected pregnant women. All participants provided written informed consent, and the study protocol adhered to the tenets of the Declaration of Helsinki. This study was reviewed and approved by the Institutional Review Board (IRB) of Gachon University Gil Hospital (IRB number: GAIRB2021-424).

### Variables

Anxiety was the dependent variables in this analysis, measured using the State Anxiety Scale of the Spielberger State-Trait Anxiety Inventory (STAI), a 20-item self-reported questionnaire. Each item was rated on a 4-point scale, yielding a summative score ranging from 20 to 80, with higher scores indicating greater levels of anxiety [12]. A score above 40 on the STAI Trait Scale was used to classify individuals as highly anxious [13]. The STAI assess both state and trait anxiety, with state anxiety items including statements such as “I am tense” and “I am worried”, and trait anxiety items including “I worry too much about things that really don’t matter” and “I am content”.

The primary independent variable was COVID-19 infection, categorized based on whether the individuals were infected during their pregnancy. Additional confounders, including demographic, socioeconomic and health-related characteristics, were also analyzed. Demographic characteristics included age, and region. Socioeconomic characteristics comprised income, marriage satisfaction, and changes due to COVID-19. Changes due to COVID-19 were categorized as “yes” or “no” in response to the question, “Has your life changed due to COVID-19?”

Health-related parameters analyzed included pre-pregnancy disease, gestation age, depression, maternal-fetal attachment, PTSD, suicidal ideation, and stress related to COVID-19. Depression was assessed using Edinburgh Postnatal Depression Scale (EPDS), a validated and reliable tool widely used to screen for depression symptoms in postpartum individuals [14]. A cutoff score of 8 was used to identify significant depression, with items such as “I have blamed myself unnecessarily when thing went wrong” included in the assessment [15]. Maternal-fetal attachment was evaluated using Maternal Fetal Attachment Scale (MFAS), which consists of 24 items divided into five subscales reflecting different dimensions of the mother-fetus relationship: (1) differentiation of self from the fetus; (2) interaction with the fetus; (3) attributing characteristics and intentions to the fetus; (4) giving of self; and (5) role taking [16]. Maternal-fetal attachment was classified using the median of the total score. PTSD symptoms were measured with the Posttraumatic Stress Disorder Checklist for DSM-5 (PCL-5), with the median score used to categorize PTSD severity. For instance, items such as “repeated, disturbing, and unwanted memories of the stressful experience” were included in the assessment. Suicidal ideation was assessed using the Beck Scale for Suicide Ideation (SSI), which is commonly used to evaluate current suicide ideation. A total score above 6 was used to indicate high levels of suicide ideation, including objective circumstances related to suicide attempts [17]. Stress related to COVID-19 was determined based on responses to the question: “Are you stressed out by COVID-19?”.

### Statistical analysis

The Chi-square test was employed to analyze the general characteristics of the study population. To compare differences between pregnant women who had COVID-19 infection and those who did not, the t-test and ANOVA were utilized. Multiple logistic regression analysis was conducted to examine the association between COVID-19 infection and anxiety among pregnant women. The results are reported as odds ratio (OR) with corresponding confidence interval (CI). Statistical significance was set at *p*-value <0.05. The C-statistic, equivalent to the area under the ROC curve, was used to evaluate discrimination and accuracy, with higher C-statistics values indicating better prediction accuracy and values closer to 1 representing the best-fitting model [18,19]. All data analyses were performed using SAS 9.4 software (SAS, Inc., Cary, NC, USA).

## Results

Table 1 presents the general characteristics of the study population. Of the 97 participants, 50 women (51.5%) experienced anxiety, and 48 (49.5%) were infected with COVID-19. The relationship between COVID-19 infection and anxiety was statistically significant, with 31 women (64.6%) who were COVID-19 positive experiencing anxiety. Table 2 shows the mean and Standard Deviation (SD) of various mental health outcomes according to COVID-19 infection status. The mean anxiety score was significantly higher in COVID-19 infected pregnant women compared to non-infected pregnant women (Infected: Mean = 46.35, SD = 13.85; Non-infected: Mean = 39.59, SD = 10.58, p-value:0.008). Additionally, depression, PTSD, and suicidal ideation were significantly higher in infected women than in non-infected women. However, there was no significant difference in maternal-fetal attachments between COVID-19 infected and non-infected women.

**Table 1:** General characteristics of study population.

Variables		Total (N = 97)		Anxiety				P-value
				Yes		No		
		N	(%)	N	(%)	N	(%)	
**COVID-19 infection**								0.0110
	Yes	48	(49.5)	31	(64.6)	17	(35.4)	
	No	49	(50.5)	19	(38.8)	30	(61.2)	
**Depression**								<0.0001
	Yes	47	(48.5)	40	(85.1)	7	(14.9)	
	No	50	(51.5)	10	(20.0)	40	(80.0)	
**Maternal-Fetal Attachment**								0.0836
	Low	49	(50.5)	21	(42.9)	28	(57.1)	
	High	48	(49.5)	29	(60.4)	19	(39.6)	
**Posttraumatic stress disorder**								<0.0001
	Yes	48	(49.5)	39	(81.3)	9	(18.8)	
	No	49	(50.5)	11	(22.4)	38	(77.6)	
**Suicidal Ideation**								<0.0001
	Yes	25	(25.8)	22	(88.0)	3	(12.0)	
	No	72	(74.2)	28	(38.9)	44	(61.1)	
**Age (years)**								0.4824
	Under 30	13	(13.4)	5	(38.5)	8	(61.5)	
	30–35	42	(43.3)	21	(50.0)	21	(50.0)	
	Over 35	42	(43.3)	24	(57.1)	18	(42.9)	
**Region**								0.5159
	Metropolitan area	34	(35.1)	16	(47.1)	18	(52.9)	
	Rural area	63	(64.9)	34	(54.0)	29	(46.0)	
**Income**								0.4340
	Low	28	(28.9)	17	(60.7)	11	(39.3)	
	Middle	36	(37.1)	16	(44.4)	20	(55.6)	
	High	33	(34.0)	17	(51.5)	16	(48.5)	
**Marriage Satisfaction**								0.0015
	Satisfied	63	(64.9)	25	(39.7)	38	(60.3)	
	Dissatisfied	34	(35.1)	25	(73.5)	9	(26.5)	
**Gestational age**								0.7188
	Under 20	19	(19.6)	8	(42.1)	11	(57.9)	
	21–30	23	(23.7)	11	(47.8)	12	(52.2)	
	31–36	19	(19.6)	11	(57.9)	8	(42.1)	
	Over 36	36	(37.1)	20	(55.6)	16	(44.4)	
**Disease before Pregnancy**								0.5086
	More than 1	17	(17.5)	10	(58.8)	7	(41.2)	
	None	80	(82.5)	40	(50.0)	40	(50.0)	
**Changes due to COVID-19**								0.1982
	Yes	62	(63.9)	35	(56.5)	27	(43.5)	
	No	35	(36.1)	15	(42.9)	20	(57.1)	
**Stress Level due to COVID-19**								<0.0001
	Yes	65	(67.0)	39	(60.0)	26	(40.0)	
	No	32	(33.0)	11	(34.4)	21	(65.6)	
**TOTAL**		**97**	**(100.0)**	**50**	**(51.5)**	**47**	**(48.5)**	

**Table 2:** Results of mean and SD according to COVID-19 infection.

Variables	COVID-19 infection							*P*-value
	Yes				No			
	Mean		SD		Mean		SD	
**Anxiety**	46.35	±	13.85		39.59	±	10.58	0.008
**Depression**	10.33	±	7.14		7.35	±	5.35	0.022
**Maternal-Fetal Attachment**	67.54	±	14.34		64.86	±	13.52	0.345
**Posttraumatic stress disorder**	40.70	±	20.75		32.22	±	12.38	0.017
**Suicidal Ideation**	6.88	±	8.84		3.22	±	4.73	0.014

Table 3 presents the association between COVID-19 infection and anxiety among pregnant women. Model 1 shows the unadjusted OR of anxiety for COVID-19 infected women compared to non-infected women. Those infected with COVID-19 during their pregnancy had significantly higher odds of anxiety, compared to non-infected women (unadjusted OR = 2.88; 95% CI = 1.26–6.57). The model fit, as measured by C-statistics, was 0.63. Model 2, which included additional mental health-related variables, showed an improved model fit with a C-statistics of 0.91. Model 3, adjusted for all covariates, demonstrated the best fit with a C-statistics of 0.94. In this fully adjusted model, COVID-19 infected women had significantly higher odds of anxiety compared to non-infected women (adjusted OR = 9.37; 95% CI = 1.39–63.12). Additionally, women who were depressed had higher odds of anxiety compared to non-depressed women (adjusted OR = 9.82, 95% CI = 1.81–53.24). However, there was no significant association between high maternal-fetal attachment and anxiety compared to those with low maternal-fetal attachment. Pregnant women dissatisfied with their marriage had higher odds of anxiety than those who were satisfied (adjusted OR = 9.61 95% CI = 1.48–62.32).

**Table 3:** Factors associated with anxiety.

Variables		Anxiety													
		Model 1					Model 2					Model 3			
		OR	95% CI				Adjusted OR	95% CI				Adjusted OR	95% CI		
*Model fit*															
	C-statistics	**0.63**					**0.91**					**0.94**			
**COVID-19 infection**															
	Yes	2.88	(1.26	–	6.57)		3.76	(1.09	–	13.04)		9.37	(1.39	–	63.12)
	No	1.00					1.00					1.00			
**Depression**															
	Yes						9.16	(2.58	–	32.55)		9.82	(1.81	–	53.24)
	No						1.00					1.00			
**Maternal-Fetal Attachment**															
	Low						1.00					1.00			
	High						0.47	(0.14	–	1.56)		0.50	(0.09	–	2.86)
	Yes						8.24	(2.14	–	31.71)		18.13	(2.44	–	134.76)
	No						1.00					1.00			
**Suicidal Ideation**															
	Yes						1.20	(0.21	–	6.94)		0.76	(0.08	–	7.25)
	No						1.00					1.00			
**Age (years)**															
	Under 30											1.00			
	30–35											1.48	(0.09	–	24.98)
	Over 35											3.47	(0.17	–	72.14)
**Region**															
	Metropolitan area											1.00			
	Rural area											2.97	(0.54	–	16.29)
**Income**															
	Low											1.36	(0.20	–	9.12)
	Middle											1.89	(0.30	–	11.78)
	High											1.00			
**Marriage Satisfaction**															
	Satisfied											1.00			
	Dissatisfied											9.61	(1.48	–	62.32)
**Gestational age**															
	Under 20											0.89	(0.10	–	7.91)
	21–30											0.94	(0.15	–	5.95)
	31–36											1.05	(0.11	–	10.24)
	Over 36											1.00			
**Disease before Pregnancy**															
	More than 1											1.25	(0.22	–	7.09)
	None											1.00			
**Changes due to COVID-19**															
	Yes											1.06	(0.25	–	4.57)
	No											1.00			
**Stress Level due to COVID-19**															
	Yes											1.42	(0.26	–	7.87)
	No											1.00			

## Discussion

With the uncertainty surrounding COVID-19 infection and the isolation caused by social distancing, many individuals have experienced increased anxiety and depression. For pregnant women, these impacts can be more pronounced due to concerns about fetal health. This study investigated how COVID-19 infection affected the mental health of pregnant women, with a particular focus on anxiety. Our findings indicate that pregnant women infected with COVID-19 exhibited higher anxiety compared to those who were not infected. Additionally, the mean levels of maternal depression and PTSD were higher in the infected group, although there was no significant difference in fetal attachment between the two groups. The symptoms of anxiety, maternal depression, fetal attachment, and PTSD were evaluated using validated screening tools, ensuring reliable assessment.

Based on our results, pregnant women infected with COVID-19 had higher mean anxiety score compared to non-infected pregnant women. Additionally, levels of depression, PTSD, and suicidal ideation were significantly higher among the infected group. The COVID-19 pandemic has been a significant life stressor, contributing to psychological distress, and this stress is a major risk factor for mental health problems in perinatal women [20]. Approximately 10~15% of all pregnant women experience emotional changes that increase the risk of anxiety and depression, potentially adversely affecting both the mothers and their developing fetuses [21]. However, our results also indicate no significant difference in maternal-fetal attachment between the infected and non-infected groups. Maternal-fetal attachment is a critical indicator of the well-being of pregnant women and their fetuses [20]. Interestingly, previous studies have shown that health worries, anxiety, and resilience related to COVID-19 can act as protective factors associated with stronger maternal-fetal attachment [22].

Pregnancy is a particularly vulnerable period, and studies have shown that pregnant women experience higher levels of distress during infectious disease outbreaks [21]. Our study indicates that pregnant women infected with COVID-19 are more likely to experience anxiety compared to those who are not infected. Similar psychological responses were observed during the 2003 Severe Acute Respiratory Syndrome (SARS) outbreak, where concerns about infection, transmission to the fetus, and the risk associated with drug treatments heightened anxiety among pregnant women [23,24]. This anxiety is driven not only by medical risk but also by the social consequences of the pandemic, such as social distancing, isolation, and reduced social support [22]. Furthermore, our findings shows that pregnant women with pre-existing PTSD or lower marital satisfaction experience higher levels of anxiety. These results highlight that both external factors, like societal disruptions, and personal factors, such as pre-existing mental health conditions and relationship satisfaction, significantly influence the anxiety levels of pregnant women during infectious disease outbreaks [21,25].

This study provides valuable insights into the unique regional context of the Incheon area, taking into account its diverse population and varied regional characteristics. Incheon, as both a metropolitan area and a region encompassing various islands, reflects a wide range of socioeconomic characteristics. These findings can serve as foundational data for understanding the impact of such diversity on maternal mental health in South Korea. Managing mental health during pregnancy is critically important, particularly in the context of infectious disease like COVID-19. The mental health issues faced by mothers due to the pandemic could exacerbate existing social and economic inequalities [26]. Given the potential for future pandemics, the findings of this study highlight the need for proactive mental health management and policy development. Addressing these challenges is necessary to better prepare for and mitigate the impacts of similar public health crisis in the future.

It should be noted that this study had several limitations. First, the results are based on self-reporting. However, most variables utilized validated scales, and specifically for mental health-related variables, using self-reported scores based on these validated scales is considered effective. Although some items may be subject to recall bias, these were measured with the assistance of medical staff, and we believe that any bias is minimal due to the diligent record-keeping by the pregnant women [27]. Additionally, the characteristics of the COVID-19 infected women and non-infected women do not perfectly match, which may result from individual differences. However, due to the simultaneous nature of COVID-19 pandemic, and considering that the participants were pregnant women who visited the same hospital, the impact is expected to be small. Furthermore, the confidence intervals are wide due to the small sample size, which can be attributed to the relatively low incidence of COVID-19 in the population and challenges in recruiting participants [28]. Nevertheless, considering the scarcity of research on pregnant women infected with COVID-19, these results could be significant. Further research should aim to include a larger sample size to enhance the robustness of the findings. Despite these limitations, certain strengths of this study are noteworthy. This study focuses on pregnant women infected with COVID-19, a population that remains under-researched due to their vulnerability. These results can provide meaningful insights and serve as fundamental data for further research on managing the mental health of pregnant women in the context of other infectious diseases. Additionally, the inclusion of various validated mental health tools in this study offers valuable insights for health policy development and enable international comparisons with other countries, helping to create a solid foundation for policies aimed at supporting maternal mental health during pandemics.

## Conclusion

The current study identifies a significant association between COVID-19 infection and mental health outcomes among pregnant women, with a particular focus on maternal anxiety. By employing validated mental health assessment tools administered by trained nurses, this study provides a comprehensive evaluation of the impact of COVID-19 infection on maternal mental health. The findings reveal that pregnant women infected with COVID-19 experience significantly higher levels of anxiety compared to those who were not infected. Additionally, the mean scores for maternal depression and PTSD were notably higher in the COVID-19 infected group. However, there was no significant difference in fetal attachment between two groups. These adverse mental health outcomes are likely driven by concerns about fetal health. This research underscores the urgent need for integrated physical and psychological care for pregnant women affected by COVID-19. By highlighting the importance of mental health management, our study offers essential insights for health policy development and clinical practice. The use of validated mental health tools not only enhances our understanding but also supports international comparisons, which is vital for improving outcomes for pregnant women and their children.
